# Antioxidant Properties of the Mung Bean Flavonoids on Alleviating Heat Stress

**DOI:** 10.1371/journal.pone.0021071

**Published:** 2011-06-10

**Authors:** Dongdong Cao, He Li, Jianyong Yi, Jingjing Zhang, Huilian Che, Jiankang Cao, Liu Yang, Chunqiu Zhu, Weibo Jiang

**Affiliations:** College of Food Science and Nutritional Engineering, China Agricultural University, Beijing, People's Republic of China; Université Joseph Fourier, France

## Abstract

**Background:**

It is a widespread belief in Asian countries that mung bean soup (MBS) may afford a protective effect against heat stress. Lack of evidence supports MBS conferring a benefit in addition to water.

**Results:**

Here we show that vitexin and isovitexin are the major antioxidant components in mungbean (more than 96% of them existing in the bean seed coat), and both of them could be absorbed via gavage into rat plasma. In the plasma of rats fed with mungbean coat extract before or after exposure to heat stress, the levels of malonaldehyde and activities of lactate dehydrogenase and nitric oxide synthase were remarkably reduced; the levels of total antioxidant capacity and glutathione (a quantitative assessment of oxidative stress) were significantly enhanced.

**Conclusions:**

Our results demonstrate that MBS can play additional roles to prevent heat stress injury. Characterization of the mechanisms underlying mungbean beneficial effects should help in the design of diet therapy strategies to alleviate heat stress, as well as provide reference for searching natural medicines against oxidative stress induced diseases.

## Introduction

In the summer hot days, “mung bean soup” (MBS, the supernatant in a light soup) prepared at home or offered freely in canteens is the most popular beverage in China and many other Asian countries. It is best-known that MBS can help people avoid and eliminate heatstroke. However, the very existence of the function of MBS on heatstroke has been questioned as adequate intake of water alone can also be helpful to prevent heatstroke. Identification of a specific property of MBS that attributes to alleviating heat stress could provide scientific supports to reveal the mechanisms implicated in health benefits of the traditional Asian diet.

Generally, heat stress is characterized by dehydration, which is caused by both inadequate fluid intake and excessive fluid loss primarily through sweating [1]. Meanwhile, heat stress may result in increased metabolic rate to meet the needs of active tissues and maximize energy yield [2], which is usually accompanied with excessive formation of reactive oxygen species, thus causing a redox imbalance between free radicals and antioxidant defense system [3].

Previous studies have demonstrated that flavonoids have specific properties of health benefits [4], such as enhancing plasma antioxidant activity [5] and preventing cardiovascular diseases [6], cancer etc [7]. Previous study showed that mungbean contained some kinds of natural antioxidants, such as flavonoids [8]. However, little information is available regarding the effects of flavonoids from food against heat stress. Identification of a specific property of MBS that attributes to alleviating heat stress could provide scientific supports to reveal the mechanisms implicated in health benefits of the traditional Asian diet.

The objective of this study was to characterize the mechanisms underlying effects of MBS against heat stress injury and identify the major components in MBS that accounts for the beneficial effect. Our result suggested that flavonoids from mung bean seed coat played a key role to prevent oxidative injury caused by heat stress.

## Materials and Methods

### Chemicals

2,2-Diphenyl–1-picrylhydrazyl (DPPH), 2,2′-azino-bis-(3-ethylbenzothiazoline-6-sulfonic acid) diammonium salt (ABTS), and 6-hydroxy-2,5,7,8-tetramethyl chroman-2-carboxylic acid (Trolox, a hydrophilic derivative of tocopherol) were purchased from Sigma-Aldrich Chemical Co. (United States), methanol and acetic acid of HPLC grade were from Fluka Chemical Co. (Germany), Potassium persulfate, and other reagents were analytical grade.

### Preparation of mungbean soup and the reference materials

Mung bean (cv. Ming) was obtained from a local market in Beijing, China. Mung bean soup (MBS) was prepared according to a traditional cooking method in China. 10 g mung bean with 500 mL deionized water (DW) was boiled for 20 min, then filtered though a filter paper (Whatman No. 1). The filtrate was added with DW to a volume of 500 mL, which was used as standard MBS for the subsequent analysis.

For analysis of antioxidant contributions of the mung bean coat, 10 g mung bean were covered with wet tissue paper and sealed in a petri plate at room temperature (22–25°C) for 6–8 h, then carefully dissected the coat. A mung bean coat soup and a coat-free MBS were prepared by boiling the collected coat or the coat free seed as described above.

Green tea (cv. Longjing) was obtained from a local market in Beijing, China. The tea soup was prepared by steeping 2 g green tea into 400 ml of 80°C DW in a beaker, then filtered though a filter paper after it cooling to room temperature (25°C, in about 50 min). The filtrate was added with DW to a volume of 400 mL, then used for the analysis.

### HPLC analysis

A HPLC system (LC- Prominence -20AT and SPD-M20A, Shimadzu Co., Japan) equipped with an analytical column C18 (Shim–pack VP-ODS 15 cm×4.6 mm ID, 5 µm, Shimadzu Co., Japan) and diode array detector was employed and performed at 30°C for analysis of the functional components in mung bean. The absorption spectra were first recorded from 210–600 nm for all peaks; UV absorbance at 280 nm was used for monitoring various phenolic compounds; the maximum UV absorbance of the sample was at 337 nm, which was used for quantifying the flavonoid. One injection volume of sample was 10 µL. Elution was done using two solvent gradients: solvent A (1% acetic acid in DDW, v/v) and solvent B (1% acetic acid in methanol, v/v). Elution was carried out at a flow rate of 1 mL/min. The gradient program (expressed as percentages of solvent B) was as follows: 0–10 min, 10–35%B; 11–25 min, 35–42%B; 26–35 min, 42–75%B; 36–40 min, 75%B; 41–45 min, 75–10%B; 46–50 min, 10%B.

### Semi-preparative HPLC separation

To obtain enough amount of the flavonoids for the subsequent analysis, semi-preparative HPLC with a column C_18_ (capcell pak UG 25 cm×10 mm ID, 10 µm, Shiseido Co., Japan) was used and performed at 30°C. The mobile phase consisted of 35% methanol was used under isocratic condition at a flow rate of 4 mL/min. One injection volume of sample was 100 µL. UV absorbance was maintained at 337 nm. According to retention time of the compound-1 and compound-2, their outflows were carefully collected. This performance was repeated for 10 times. The collected fractions were first concentrated by rotatory evaporating, and then lyophilized (LGJ-10, Henan Brother Co. Ltd., China) for 24 h. Purity of the compound-1 or the compound-2 was examined by HPLC at UV absorbance wavelength of 230, 260, 280, 300, 320, 337, 360 nm. Sequentially, 7 chromatographs were generated for quantifying compounds in the samples by peak area normalization method. The results indicated that purity of the compound-1 was more than 96% and purity of the compound-2 was more than 95% with the same statistical method.

### Preparation of mung bean coat extracts

Mung bean seed coat was obtained from a factory of mung bean starch processing in Jilin-province in China, and carefully handled to remove impurities. Then the mung bean coat was used for the subsequent experiments. The mung bean coat extract (MBCE) was prepared by boiling 10 g mung bean coat with 100 mL DW for 20 min. Thereafter the MBCE was filtered though a filter paper (Whatman No. 1). The filter residue with a small amount of DW was re-filtered. The 1^st^ filtrate was diluted with the 2^nd^ filtrate to a volume of 100 mL, which was then used as the standard MBCE.

### Determination of DPPH radical scavenging capacity

The antioxidant activity was determined by DPPH assay according to previous study [9] with some modifications. Aliquot of 200 µL sample mixed with 3.8 mL DPPH solution (200 µM in methanol) was incubated in dark at room temperature for 60 min, then its absorbance at 517 nm was measured by a spectrophotometer. Scavenging ability of the sample to DPPH radical was determined according to the following equation:
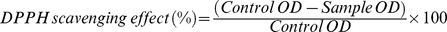



### Determination of ABTS·^+^ radical scavenging capacity

The experiments were carried out according to the procedure of ABTS·^+^ scavenging assay as described previously [10]. Trolox, a water soluble analogue of vitamin E, was used as a reference standard. A standard curve was plotted based on measuring the reduction in absorbance of the ABTS·^+^ solution against a concentration range between 100 µM and 600 µM of trolox. ABTS·^+^ scavenging capacity of sample was expressed as trolox equivalent antioxidant capacity, which represents that the concentration of trolox solution had the same antioxidant capacity as the sample.

### Determination of hydroxyl radical (OH·) and superoxide anion radical (O_2_^−^) scavenging capacities

The capacities of scavenging OH· and O_2_·^−^ were estimated by reagent kits (NanJing JianCheng Bio Inst, Nanjing, China), respectively.

### Rats and serum samples preparation

Male Wistar rats (body weight: 190–210 g) were provided by Animal Department of Academy of Military Medical Sciences. Rats were housed in polypropylene cages, in a temperature-controlled room (23±2°C) at relative humidity (60±75%) with a 12 hour dark/light cycle. The animals had free access to water and food. All experimental protocols were approved by the Animal Management Rules of the Ministry of Health of the People's Republic of China (documentation Number 55, 2001, Ministry of Health of PR China). All surgery was performed under anesthesia, and all efforts were made to minimize suffering.

For serum preparation, the rats were anaesthetized with diethyl ether and blood samples were collected by orbital venous plexus approach (bleeding). The serum samples were separated and stored at −20°C for the relevant assays.

### Analyzing the pharmacokinetics of the flavonoids in rat

After in fasting for 12 h,each of the 6 rats was fed with 2 mL of standard MBCE (equal to 1000 mg mung bean coat/kg rat) via gavage. The rats were anaesthetized with diethyl ether, then blood samples (about 0.5 mL per sample) were collected with anti-coagulation tube (BD Co., USA) at 20, 40, 60, 90 and 150 min after the MBCE gavage.

For analysis flavonoids level in the rat plasma samples, 200 µL the rat plasma sample with 800 µL methanol were mixed by vortexing for 2 min, then centrifuged at 8000 *g* for10 min at 4°C. The supernatant was collected and evaporated at 50°C to dryness under nitrogen. The residue was re-dissolved in 100 µL of 35% methanol before the HPLC analysis. The rest of the plasma sample was used for subsequent analysis.

### Analyzing the effects of MBCE on rat exposed to heat stress

The rats were adapted to external environment for 7 days, which consisted of a swimming exercise in water cells (40 cm×15 cm×50 cm) at 35±1°C for 5 min every other day. The swimming cells were kept with running water to wash out feces. After the adaption, the rats in fasting for 12 h were randomly divided into 9 groups (6 rats each group), then each of the rats was fed with MBCE or DW via gavage as follows.

Group Ι, 2 ml of DW and served as untreated control; Group II, 2 mL of DW and served as control of unfed with MBCE; Group III, per rat 2 mL of 10% MBCE (equal to 100 mg mung bean coat/Kg rat); Group IV, 2 mL of 20% MBCE (200 mg/Kg); Group V 2 mL of 40% MBCE (400 mg/Kg).

After the gavages for 10 min, the rats of GroupII, III, IV and Vwere exposed to heat stress in swimming cells at 40±1°C for 30 min. Each swimming cell only had one rat at the same time to avoid disturbing each other. Blood samples were collected immediately after the heat-treatment.

The rats of GroupVI, VII, VIII or IX were first exposed to heat stress as described above, then immediately fed with MBCE or DW via gavage as the rats of GroupII, III, IV or IX respectively. Blood samples were collected at 40 min after the gavages.

### Biochemical assays of the rat serum

The total antioxidant capacity (T-AOC), the activities of lactate dehydrogenase (LDH) and nitric oxide synthase (NOS), contents of glutathione (GSH) and malonaldehyde (MDA) were estimated by reagent kits (NanJing JianCheng Bio Inst, Nanjing, China),respectively. The principles of these kits are briefly described as follows.

T-AOC was determined by catalyzing ferric reducing ability of the sample [11]. This assay was determined based on the reduction of Fe^3+^ -TPTZ to a blue coloured Fe^2+^ -TPTZ. The increase in the absorbance was determined by a spectrophotometer at 593 nm. The T-AOC value was expressed as U/mL serum or plasma, which is equal to increase of 0.01 OD unit per mL serum per minute in the measuring system.

LDH activity was determined by catalyzing lactic acid into pyroracemic acid and in turn reacting with 2, 4-dinitrophenylhydrazine to form a brown complex monitored at 440 nm. Values were expressed by international units (IU) per liter serum.

NOS activity was determined by catalyzing L-arginine and molecular oxygen into nitric oxide (NO), which reacts with nucleophile to form the complex monitored at 530 nm. Values were expressed as U/mL serum, which is equal to formation of 1 nmol NO per mL serum per minute in the measuring system.

MDA was determined by its reaction with TBA to form thiobarbituric acid-reactive substances (TBARS) that detected at 532 nm. Content of MDA was expressed as nmol MDA /ml serum.

GSH was measured in terms of its reaction with 5, 5-dithio-bis-nitrobenzoic acid (DTNB) for color development that monitored at 412 nm. Content of GSH was expressed as mg GSH /L serum.

### Statistical analysis

Statistical analyses were performed with SPSS 11.5 for windows. All the data were subjected to one-way analysis of variance (ANONA) and the significance of difference between groups was calculated by least significant difference (LSD) test. *P*<0.05 indicated statistical significance.

## Results

### Antioxidant capacity of mung bean soup

**Figure 1:**
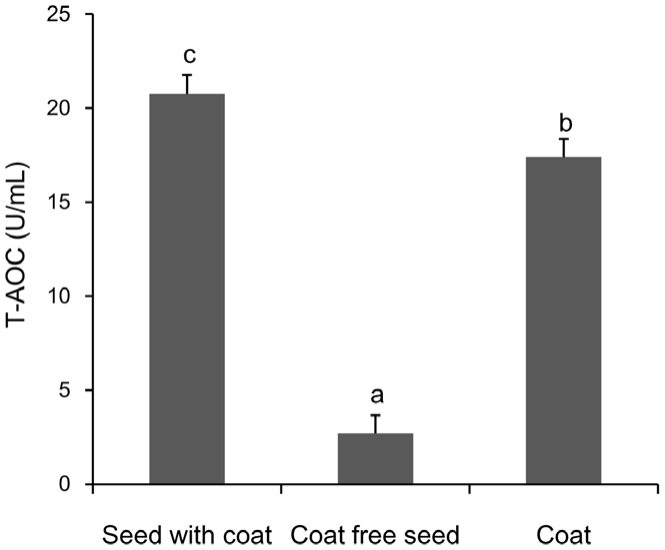
Antioxidant capacity of mung bean soup. The total antioxidant activity (T-AOC) of MBS and the bean cotyledon soup made from the same quantity of mung bean. Data represent mean ± s.d.; n = 3.

We measured the total antioxidant activity (T-AOC) of MBS and found that the bean seed coat soup had a much higher T-AOC value than the bean cotyledon soup (*P*<0.05) made from the same quantity of mung bean (Fig. 1). The coat contributes about 87% of the mungbean T-AOC.

**Figure 2:**
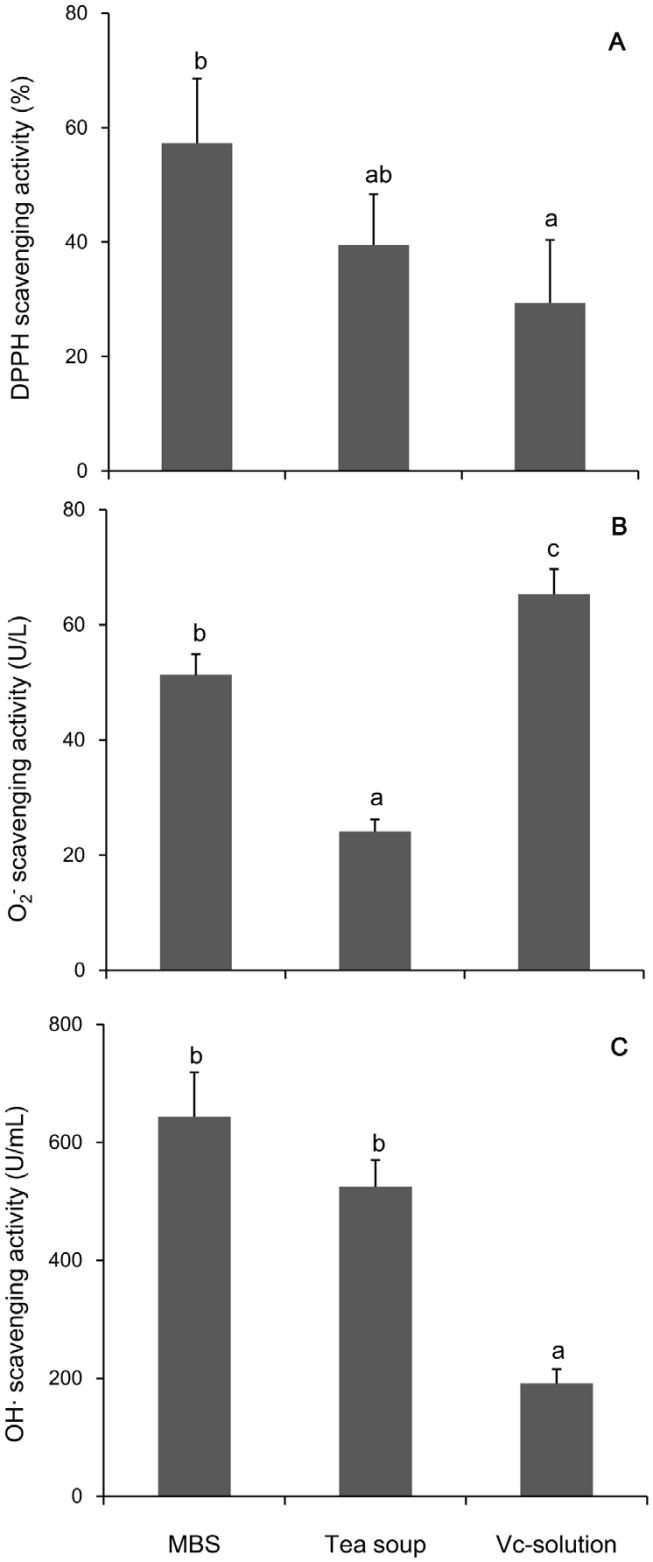
Comparison on antioxidant capacity. Comparing the MBS (in d.m. 20 mg /mL) with a tea soup (in d.m. 5 mg/ml) and a vitamin-C solution (0.15 mg/ml) on DPPH·-SA (A), O_2_·-SA (B) and OH·-SA (C). Data represent mean ± s.d.; n = 3.

Comparing the MBS (20 mg/ml) with a tea soup (5 mg/ml) and a vitamin-C solution (0.15 mg/ml) on antioxidant capacity, we found that DPPH scavenging activity (SA) of the MBS was approximately 145% that of the tea soup or 195% that of the vitamin-C solution (Fig. 2A), which means that the DPPH-SA of 100 g mungbean is equivalent to that of 36.3 g dried green tea or 1462 mg vitamin-C. Similarly, 100 g mungbean is equivalent to 53.2 g tea or 589 mg vitamin-C on O_2̇_-SA [12] (Fig. 2B), and equivalent to 30.6 g dried tea or 2521 mg vitamin-C on OH·-SA [12] (Fig. 2C).

### Attribution of the major flavonoids in the total antioxidant capacity of MBS

**Figure 3:**
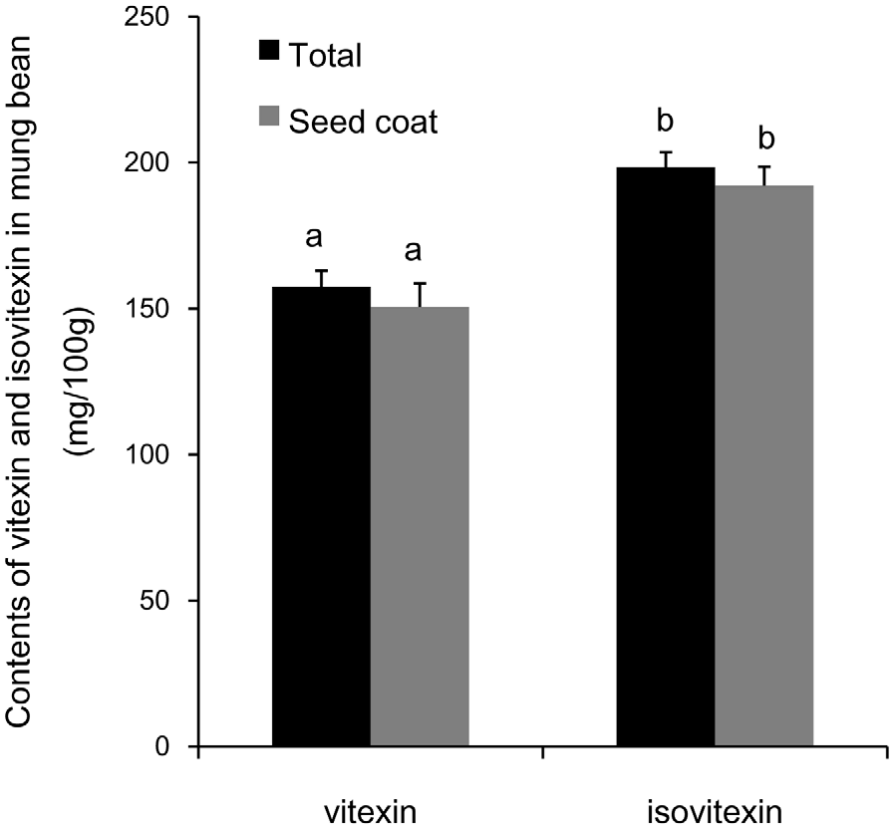
Contents of vitexin and isovitexin in mungbean. Contents of vitexin and isovitexin in mungbean were quantitatively measured by the HPLC system. A model solution was prepared according to levels of vitexin (157.5 mg/100 g) and isovitexin (198.5 mg/100 g) in mungbean with the purified vitexin and isovitexin by a semi-prepared HPLC system. Data represent mean ± s.d.; n = 3.

Previous study has demonstrated that the two major flavonoids in MBS was vitexin and isovitexin [8].Quantitative analysis showed that the contents of vitexin and isovitexin in mungbean were 157.5 and 198.5 mg/100 g (Fig. 3), the seed coat contributed to 95.6% of the total vitexin and 96.8% of the total isovitexin in the mungbean.

**Figure 4:**
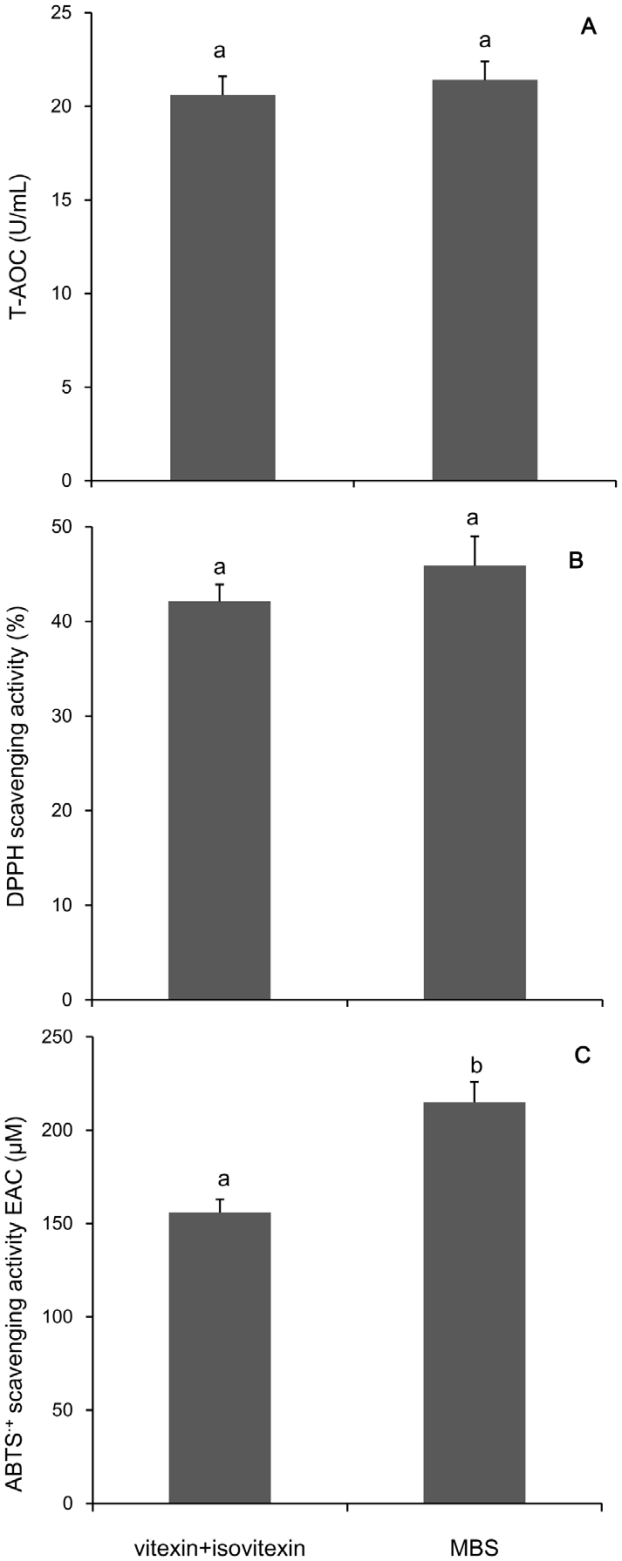
Attribution of vitexin and isovitexin in the antioxidant capacity of MBS. Vitexin and isovitexin purified by a semi-prepared HPLC system were analyzed on T-AOC (A), DPPH•-SA (B), ABTS·+-SA (C). Data represent mean ± s.d.; n = 3.

In order to learn how vitexin and isovitexin might contribute to the antioxidant capacity in MBS, a modeling solution containing the purified vitexin and isovitexin at the same levels as present in MBS was prepared. The antioxidant capacity of modeling solution was evaluated by measuring its T-AOC, DPPH or ABTS·^+^-SA. Our results indicated that the two flavonoids from mungbean coat contributed 96.2% of T-AOC (Fig. 4A), 91.8% of the total DPPH-SA (Fig. 4B) or 72.5% of ABTS·^+^-SA of the MBS (Fig. 4C), respectively. These results demonstrated that vitexin and isovitexin are the major antioxidant contributors in MBS.

### Bioavailability of vitexin and isovitexin of mung bean in rat

**Figure 5:**
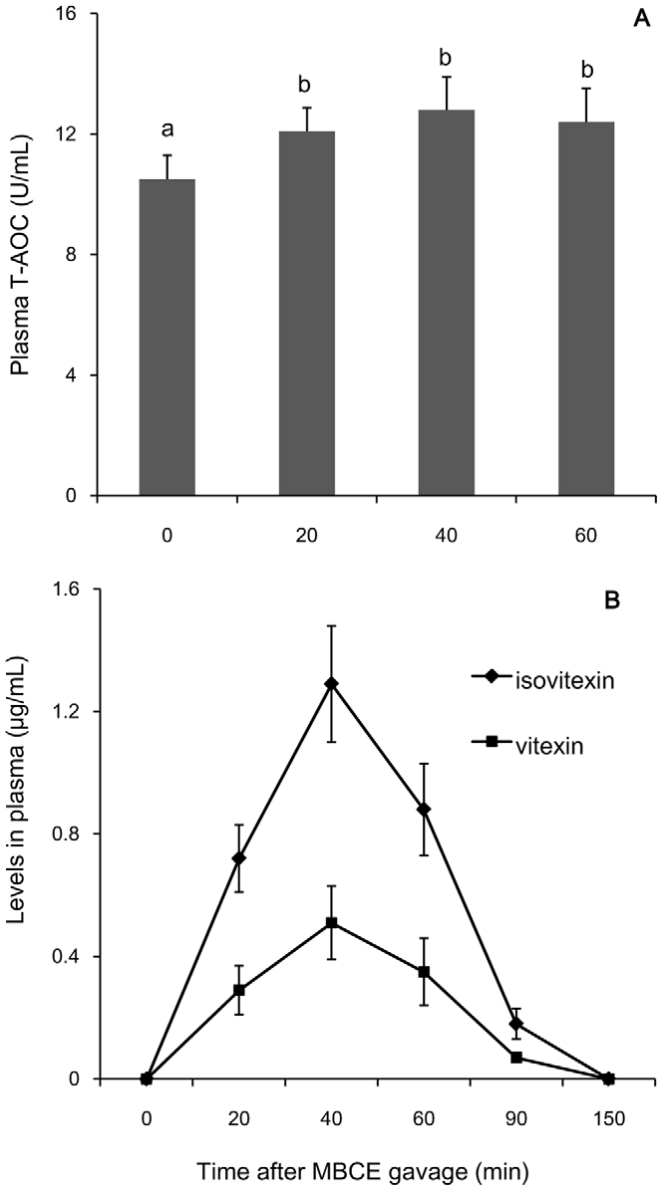
Bioavailability of vitexin and isovitexin in rat. For analyzing *in vivo* absorption of the flavonoids from MBCE and its effect on T-AOC in plasma, the rats were fed with MBCE (1000 mg/kg BW) via gavage. The plasma samples were collected at the defined times and used for measuring the T-AOC (A) and quantitative analysis of vitexin and isovitexin by HPLC (B). Data represent mean ± s.d.; n = 6.

To explore *in vivo* the properties of the flavonoids, we used rat as a model for determining effect of the MBCE on antioxidant activity in plasma and their pharmacokinetics. The levels of T-AOC in plasma were significantly enhanced by feeding the rats with MBCE (Fig. 5A, *P*<0.05). Both vitexin and isovitexin in the rat plasma could be observed by HPLC analysis at 20 min after MBCE gavage, and reached to their peaks in about 40 min after the gavage, thereafter, declined rapidly (Fig. 5B). Levels of isovitexin were much higher than those of vitexin in the plasma (*P*<0.05). Taking account of that the isovitexin content just 26% higher than vitexin in the mungbean (Fig. 3), and level of isovitexin was 148.3%, 152.9% or 151.4% higher than that of vitexin at 20, 40 or 60 min after the gavage (Fig. 5B), respectively. This result may suggest that bioavailability of the isovitexin is much higher than the vitexin *in vivo*.

### Protective effects of MBS on rat against heat stress

Injury of heat stress is usually accompanied with excessive formation of reactive oxygen species [2]. It is reasonable to deduce that the enhancement of antioxidant capacity could help to preventing oxidative injury of heat stress. To verify this deduction, we further investigated the effects of MBCE on rat *in vivo* against heat stress.

**Figure 6:**
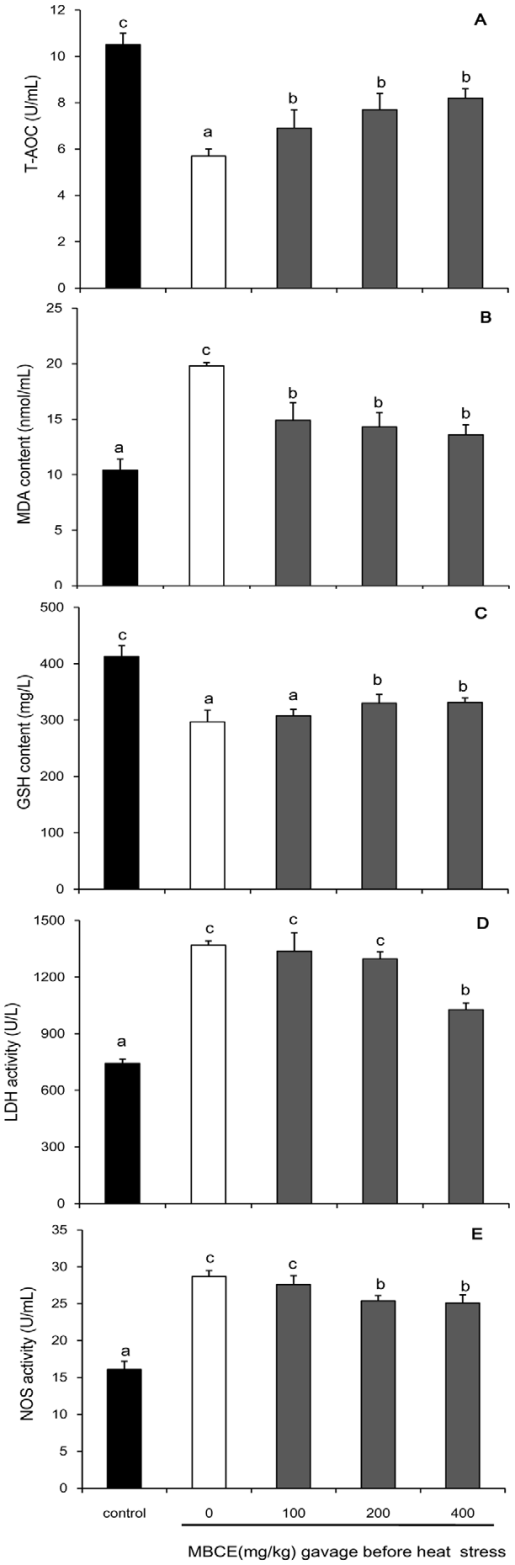
Effects of pre-administration of MBCE against heat stress in rat. For analyzing effects of MBCE against heat stress, the rats were fed with MBCE (100, 200 or 400 mg/kg BW) at 10 min before heat treatment and collected plasma samples immediately after the heat-treatment. T-AOC (A), MDA content (B), GSH content (C), LDH activity (D) and NOS activity (E) were determinate. Data represent mean ± s.d.; n = 6.

Our experiment models of heat stress by allowing the rats to swim in hot water (40±1°C for 30 min) showed that the heat exposure caused a significant decline in levels of T-AOC (45.7%) and GSH (28.2%) in the rat plasma (Fig. 6A, *P*<0.05). The heat stress induced significant increases in levels of MDA, LDH and NOS in the rat plasma (*P*<0.05).

**Figure 7:**
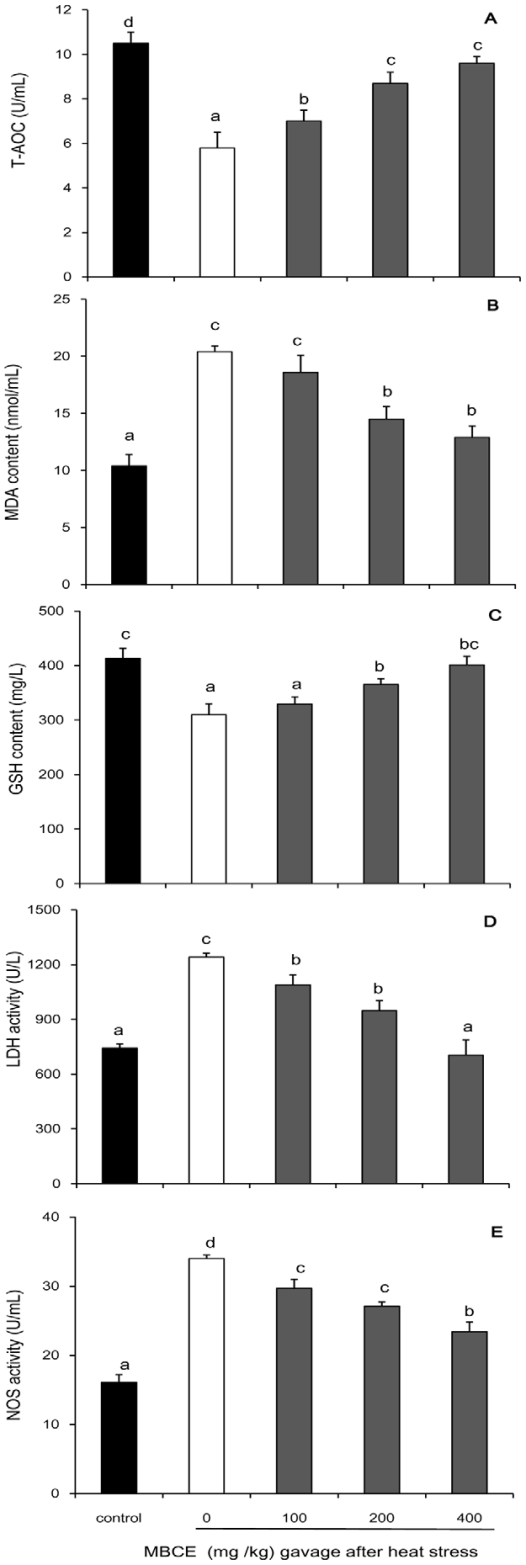
Effects of post-administration of MBCE against heat stress in rat. For analyzing effects of MBCE against heat stress, the rats were fed with MBCE (100, 200 or 400 mg/kg BW) immediately after the heat treatment and collected plasma samples at 40 min after gavage. T-AOC (A), MDA content (B), GSH content (C), LDH activity (D) and NOS activity (E) were determinate. Data represent mean ± s.d.; n = 6.

Compared with those in rats exposed to heat stress without MBCE gavage, T-AOC and GSH levels in plasma were enhanced for 35.1% and 11.2% by the MBCE gavage (200 mg/kg BW) before the rats exposure to heat stress (*P*<0.05); the pre-gavage of MBCE also induced 27.8% decrease in MDA level, 5.2% and 11.5% decreases in activities of LDH and NOS in the rat plasma (Fig. 6), respectively. Similar effects of MBCE on T-AOC, GSH, MDA, LDH and NOS in plasma were observed when the rats were fed with MBCE after exposure to heat stress (Fig. 7). Compared with those rats exposed to heat stress but not fed with MBCE, the T-AOC and GSH levels in plasma were significantly enhanced in rats fed with MBCE after exposing to heat stress (*P*<0.05); the post-gavage of MBCE also significantly lowered the levels of MDA, LDH and NOS in the rat plasma, respectively (*P*<0.05).

## Discussion

Generally speaking, intake of water can be benefit to preventing heatstroke. However, heatstroke is usually accompanied with excessive formation of reactive oxygen species (ROS), and then may cause an oxidative injury to organisms [13]. Our results strongly support the hypothesis that MBS can play an additional role to prevent injury caused by heat stress.

Studies have demonstrated that dietary intake of fruits and vegetables have specific properties of health benefits, which are usually attributed to the antioxidant ability of flavonoids and the other antioxidant components from fruits and vegetables [14]–[16]. Some populations living in desert and/or undeveloped region, particularly in wintertime have been usually lacking fruit and vegetable in their diet. However, they usually eat a lot of beans, including mung bean. Our results suggest that adequate mung bean diet can fill up to some extent the deficiency of lacking fruit and vegetable.

Vitexin and isovitexin have also been found in some other plants, such as bamboo leaves [17], and pigeonpea leaves [18] and showed their antioxidant activities *in vitro*. However, little information is known whether vitexin and isovitexin could be biologically absorbed. Our result showed that both vitexin and isovitexin could be absorbed into plasma in the rats fed with MBCE. Meanwhile, enhancement of antioxidant activity in the plasma was also observed. These results provided further evidences supporting the view that consumption of mean bean soup could prevent or reduce injury of heat stress.

Serum MDA levels is the most commonly biomarker of the involvement of free radical damage in pathologies associated to oxidative stress [19]. We showed that MDA accumulation in plasma of the rats exposed to heat stress could be reduced significantly by MBCE intragastric administration, which suggest that MBCE could prevent lipid peroxidation in the plasma.

Glutathione (GSH) can either directly scavenge free radicals or act as a substrate for glutathione peroxidase and glutathione S-transferase during the detoxification of hydrogen peroxide, lipid hydroperoxides [20], and is often used for quantitative assessment of oxidative stress [21]. Lactate dehydrogenase (LDH) and nitric oxide synthases (NOS) are also commonly used as markers of tissue damage [22], [23]. Our analysis indicated that GSH showed a remarkably higher level in MBCE treated rat than that in control. Meanwhile, activities of LDH and NOS in plasma of the MBCE treated rat were also lower than those under heat stress in control. Furthermore, the similar effects of pre- and post-administration of MBCE on heat stress were observed in our study. Except for the effects of flavonoids in MBCE, it might be also due to water and some minerals which might be present in MBCE.

In China, people usually drink the supernatant, but not eat the bean per se in MBS supplied for alleviating heat stress. This diet habit may not reduce MBS effect against heat stress, since the major antioxidant components, including vitexin and isovitexin, mainly exist in the coat and are easily leaching into the supernatant. Furthermore, without eating the bean could avoid excessive calorie intake, therefore, be good for health.

Addition to fresh fruit and vegetable, some other kinds of food are also rich in antioxidants, such as chocolate [5], red wine [24], various dried fruits and nuts etc, but none of which has ever been considered to be good for alleviating heat stress. Various beverages, such as tea, juice, carbonated drink, even beer are also considered to be good for alleviating heat stress because of water supplementation; however, drinking of these kinds of beverages could also result in intakes of calories, caffeine and/or alcohol, which may cause other health problems, particularly of large quantity of drinking. Therefore, the traditional Asian diet, mung bean soup is a kind of extraordinary food against heat stress from a scientific point of view.

In conclusion, our results show that mung bean soup is rich in vitexin and isovitexin, and can offer protection against injury of heat stress. These results demonstrated that mung bean soup can play additional roles to prevent injury caused by heat stress.
